# Antibacterial Effects of *Artemisia afra* Leaf Crude Extract Against Some Selected Multi-Antibiotic Resistant Clinical Pathogens

**DOI:** 10.4314/ejhs.v32i3.22

**Published:** 2022-05

**Authors:** Alemtsehay Beyene Haile, Tamene Milkessa Jiru

**Affiliations:** 1 Department of Biotechnology, Institute of Biotechnology, University of Gondar, Ethiopia; 2 Department of Environmental and Industrial Biotechnology, Institute of Biotechnology, University of Gondar, Ethiopia

**Keywords:** E. coli, MIC, MBC, phytochemicals, P.aeruginosa, S.pneumoniae, S.aureus

## Abstract

**Background:**

Due to improper use of antibiotics, some pathogenic bacteria that cause serious and deadly infections have become resistant to commonly used broad spectrum antibiotics. This antibiotic resistance has become major global healthcare problem. Therefore, there is an urgent need to develop novel antibacterial agents; hence, much attention has been made on medicinal plants such as Artemisia afra. Thus, the current study was aimed to evaluate the antibacterial activity of ethanolic, methanolic and n-hexane extracts of this plant leaf against four multi-antibiotic resistant clinical pathogens.

**Methods:**

Crude extracts from A.afra leaf were prepared using ethanol, methanol and n-hexane and the antibacterial effect of each extract was tested against Escherichia coli, Streptococcus pneumoniae, Pseudomonas aeruginosa and Staphylococcus aureus. In addition, minimum inhibitory concentrations (MIC) and minimum bactericidal concentrations (MBC) were evaluated.

**Results:**

Among the crude extracts, the highest zone of inhibition (25.33±0.58mm) was recorded against E. coli when methanolic extract was applied. On the other hand, the lowest inhibition was exhibited when n-hexane extract was applied against S.aureus (5.67±1.56 mm). Concerning MIC values of the different extracts, varied results were obtained. MIC value of 6.25mg/mL was recorded when methanolic extract was applied against all clinical pathogens. Moreover, both methanolic and ethanolic extracts showed MBC value of 12.5mg/mL against the four clinical pathogens. However, the methanolic extract gave MBC value of 6.25mg/mL against E. coli.

**Conclusion:**

From this study, it can be concluded that it is possible to develop and formulate of new, efficacious, less toxic and inexpensive herbal medicine from A.afra leaf extract that act against multi-antibiotic resistant clinical pathogens.

## Introduction

Medicinal plants have been used in traditional medicine practices since ancient times. Currently, there are intense pharmacological studies on drug discovery from medicinal plants. The driving force behind this is the attention given to the value of medicinal plants as potential sources of new antimicrobial compounds of therapeutic importance and drug development in order to provide indispensable physical and physiological health care (1). There are several reasons to prefer herbal medicine over modern synthetic drugs. Herbal medicine is considered to be one option to tackle multi-drug resistance problems (2). This is because herbal medicines have little side effect in contrast to chemically synthesized drugs; therefore, they are considered as less toxic (3).

Antibiotics in some individuals elicit allergenic reaction and cause immunity suppression (2). Therefore, to tackle such problems, herbal medicines are preferred. According to the world health organization (WHO), up to 80% of African people use traditional medicine (4). Similarly, in Ethiopia around 85% of the people use medicinal herbs for their primary health care (5). In recent years, the acceptance of herbal medicine as an alternative form of healthcare has increased in many socio-economic groups of the African population and phytomedicine generates income for the local community (6). In general, the WHO recommends and encourages the use of plants as effective tool for healthcare (4).

Being rich in biological diversity, Ethiopia expects direct financial profits and economic gains from its plant, animal and microbial genetic resources. Ethiopia is believed to be a home for about more than 6,500 species of plants with approximately 12% endemic (7). The country has a long history of using medicinal plants to treat a variety of human diseases (7).

*A.afra* is a member of the daisy or Asteraceae family. It is an evergreen herbaceous perennial plant with grey or green foliage leaves containing yellow florets. The plant grows from 0.6 meters to 2 meters high, at an altitude range of 3070 to 3600 meters (8). The plant is found in Southern and Eastern parts of Africa. It is the commonly used phytomedicine in South Africa (9, 10). It is a common species of the genus *Artemisia* in Africa, widely distributed from the Eastern parts of Africa with areas stretching from South Africa, to areas reaching to the North and East, as far north as Ethiopia. In South Africa for example is called “Umhlonyane”. In Ethiopia, it has different local names such “Chugughee” in Amharic, “Ariti” in Oromifa, “Kodo” in Guragigna. *A.afra* is considered a “cure-all” because of traditionally treating a wide range of illnesses with this herbal plant. The leaves of this plant contain various phenolic bioactive compounds that have antimicrobial activity (11). This medicinal plant has gained popularity in treating bacterial infections such as sore throats, ear infections and various bronchial diseases (11). Traditionally *A.afra* is also applied in treating viral infections such as measles and influenza, and parasitic infections such as malaria and intestinal parasites (11). Other ailments treated using this medicinal herb include diabetes, heartburn, bronchitis, and asthma (9,12). Moreover, recent studies have indicated that *A.afra* has potent antimicrobial activity against different pathogens including methicillin-resistant *S.aureus, Mycobacterium smegmatis* and *M. tuberculosis* (14).

Traditional remedies are the ones that are used to treat infectious and inflammatory diseases. Treatment of bacterial disease is a common problem due to the emergence of multidrug resistant bacterial strains to numerous antibiotics (15). Some of the factors that enhance infectious diseases include poor hygiene, insufficient sanitation and congested conditions (16).

Currently research revealed that, essential oils from aromatic plants are used as food preservative (17), antibacterial (18), antifungal (19), antioxidant (20), antispasmolytic (21), anti-inflammatory and anticancer activity (22). These results show good potential of essential oil replacing synthetic antibiotics which are responsible for most of an increased resistance of pathogens (23).

An increase in multi-drug resistance towards commonly used antibiotics and the absence of new antimicrobial drugs introduced into the market resulted in a need to devise new mechanisms so as to cope with infections resulting from multiple antibiotic resistant pathogenic bacteria (24, 25). Furthermore, there is a public health calamity due to the alarmingly increasing antibiotic resistance. Also, multi-drug resistance is spreading faster than the introduction of new antimicrobial compounds into clinical practice (26). The evidences of increasing multi-antibiotic resistant pathogens at an alarming pace are seen in high rates of morbidity and mortality.

The increase in antibiotic resistance and inefficacy at existing medical treatments emphasizes the need to develop new and more efficient antibacterial drugs (27). Herbal medicines can play a key role in counteracting multi-drug resistant pathogens. Therefore, screening of medicinal plants for their potential antimicrobial activity may assist in the determination of new drugs from medicinal plants. There must be proper investigation of the traditional plants in order to discover new drugs that can complement the already existing drugs. Previous studies suggested that there are antibacterial compounds in medicinal plants (28).

There is scarcity of information about the antibacterial activities of *A.afra* against multi-antibiotic resistant clinical pathogens (*E.coli, S.aureus, P.aeruginosa*, and *S.pneumoniae*) in Ethiopia. And hence this study was aimed at evaluating the antibacterial potential of this plant leaf crude extract against multi-antibiotic resistant bacterial strains.

## Methods

**Figure 1:**
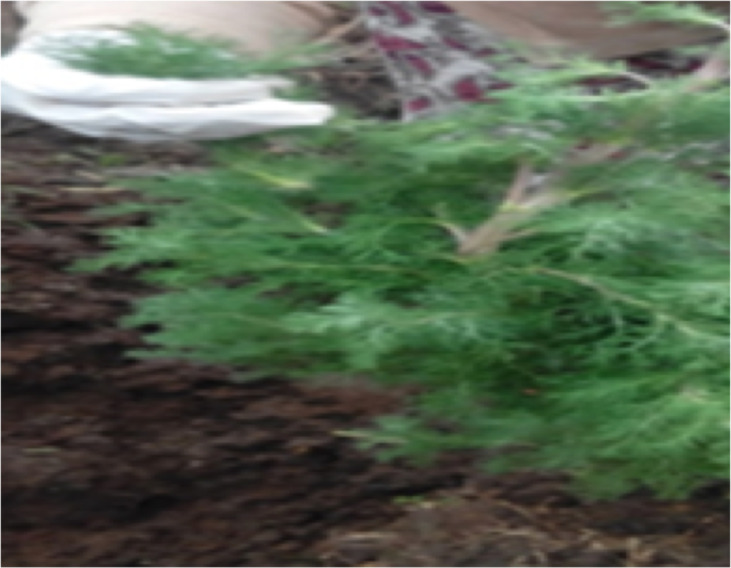
Image of *A.afra* shrub collected by Alemtsehay Beyene.

**Figure 2:**
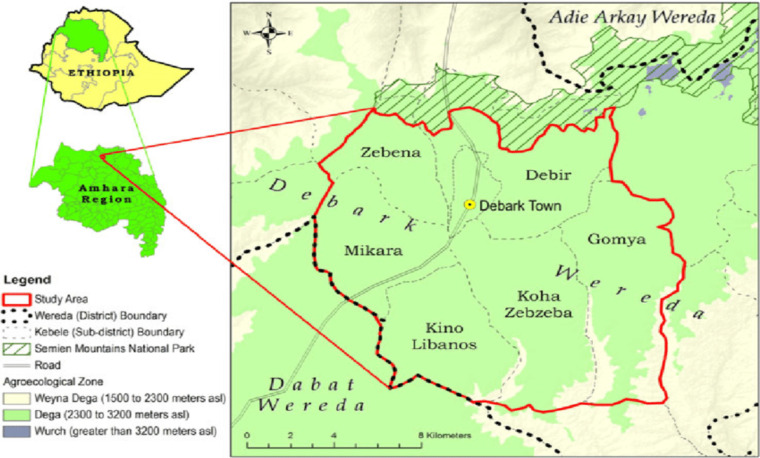
Map of study area (Debark district).

**Collection of plant materials**: In the current study, fresh leaves of *A.afra* (Figure 1) were collected from Debark district (Figure 2) from a garden place. The plant identification was further confirmed and authenticated at the Department of Biology, College of Natural and Computational Sciences, University of Gondar. Finally, voucher of the specimen with deposition number 002/ABH/2019 was deposited in the College of Natural and Computational Sciences Herbarium, University of Gondar.

**Extraction of plant material**: All experiments, including extraction of the plant material were carried out in Microbiology Laboratory, Department of Biotechnology, Institute of Biotechnology, University of Gondar. Leaves of *A.afra* were washed with tap water; air dried, chopped and powdered using a blender. To get crude solvent extract, 20g of powdered plant material was macerated with 400mL of each solvent, i.e., 99.8 % methanol, 97 % ethanol, and 99 % n-hexane in 500 mL flasks separately. The macerated mixtures were then left on the shaker for 72 h at room temperature. Extracts were filtered through Whatman filter paper No1. and concentrated under reduced pressure with a rotary evaporator at 68°C, transferred to labeled vials and allowed to stand at room temperature to permit evaporation of residual solvents. Then, the extracts were stored under refrigeration at 4°C for further studies.

**Qualitative detection of phytochemicals**: The presence of different phytochemical constituents of the leaf extracts of *A.afra* such as flavonoids, terpenoids, phenolics, cardiac glycosides, alkaloids, saponins and tannins was detected using standard procedures (29).

**Source of pathogens and inocula preparation**: The test multi-antibiotic resistant clinical pathogens were obtained from University of Gondar Comprehensive Specialized Hospital Laboratory and Microbiology unit. The isolates were *E.coli, S.aureus, P.aeruginosa* and *S.pneumoniae.* Suspension of the isolates was made in 0.9% sterile normal saline solution and adjusted to 0.5 MCFarland's standard, in order to standardize the inocula to contain about 10^8^ cfu/mL.

**Multiple antibiotic resistance (MAR) index**: The antibiotic susceptibility patterns of these test organisms were performed as per standard procedure and their MAR index was calculated by the ratio of number of antibiotics ineffective over the organisms to the number of antibiotics exposed (30). The antibiotics used in the study were ampicillin (A-10 µg), chloramphenicol (C-30 µg), ciprofloxacin (Cf-5 µg), co-trimoxazole (Co-1.25 µg), gentamycin (G-10 µg), novobiocin (Nv-30µg), tetracycline (T-30 µg), vancomycin (Va-30 µg), ceftriaxone (Ci-30 µg) and cefatizidim (Ce-30 µg).

**Antibacterial activity test using *A.afra* leaf**: The *in vitro* antibacterial assay of each leaf extract was performed using disc diffusion method as described by Kirby-Bauer (31). All the experiments were performed under sterile conditions. The Mueller Hinton agar (MHA) plates were inoculated separately with 10^8^ cfu /mL of each test pathogenic bacterial strain culture (*E.coli, P.aurguginosa, S.aureus* and *S. pneumoniae*) and evenly spread on entire surface of each plate. The sterile discs (6 mm diameter) were dipped aseptically into each extract for five minutes, while one disc was dipped into DMSO 50 % solution and served as a control. Then the discs were placed over MHA plates seeded with pathogenic bacterial culture. The plates were incubated aerobically at 37°C for 24 h and zone of inhibition was observed. Zone of growth inhibition was measured. The experiment was done in triplicate.

**Minimal inhibitory concentration**: MIC of *A.afra* leaf crude extract was done using broth dilution method. MIC was determined by serially diluting the extracts (32). The dilution ranges of crude extracts were 100 mg/mL, 50 mg/mL, 25 mg/mL, 12.5 mg/mL, 6.25 mg/mL, 3.13 mg/mL and 1.56 mg/mL. First, a 100 µL of Mueller Hinton broth (MHB) was added to the wells of a 96-well microtiter plate. Then, from each crude extract, 100µL was added into two rows of sterile 96-well microtiter plates. The wells were inoculated with 100 µL of the four multiantibiotic resistant pathogenic cultures separately at a concentration of 10^8^ cfu/mL. MHB with the inoculum only was used as growth control. The microtiter plate was incubated at 37°C for 48 h in an incubator and then 20 µL of resazurin reagent was added into each well. The plates were examined after additional 60 minute of incubation. Growth was indicated by a pink color; active bacterial cells reduce non fluorescent resazurin (blue) to the fluorescent resazurin (pink) (33).

**Minimum bactericidal concentration**: Bacterial cells from the MIC test plates were sub-cultured on the fresh MHA media. The plates were then incubated at 37°C for 24 h. Plates that did not show any visible growth of the colony on the solid agar medium after subculturing were considered as MBC. The MBC was regarded as the lowest concentration of the extracts that completely prevents the growth of any bacterial colony on a solid medium. All experiments were done in triplicates. The experimental results were expressed as mean ± standard deviation (SD) of three replicates.

## Results

**Phytochemical screening of *Artemisia afra* leaf extract**: In this study, the phytochemical screening of the crude extract of *A.afra* leaf was determined and the result showed that the studied plant contained different secondary metabolites such as cardiac glycosides, flavonoids, saponins, tannins and phenols but negative result was recorded for alkaloid as shown in Table 1 below.

**Table 1:** Phytochemical constituents of *A.afra* leaf extract

Phytochemical	Methanol	Ethanol	n-hexane
**Cardiac glycosides**	+	+	+
Saponins	+	+	+
Flavonoids	+	+	+
Tannins	+	+	+
Phenolics	+	+	+
Alkaloids	-	-	-

-: absence; +: presence

**Evaluation of selected antibiotics**: In this study, initially different antibiotics namely, ampicillin (A-10 µg), chloramphenicol (C-30 µg), ciprofloxacin (Cf-5 µg), co-trimoxazole (Co-1.25 µg), gentamycin (G-10 µg), novobiocin (Nv-30µg), tetracycline (T-30 µg), vancomycin (Va-30 µg), ceftriaxone (Ci-30 µg) and cefatizidim (Ce-30 µg) were tested against four clinical pathogens that were grown on MHA; however, none of these antibiotics were able to inhibit the growth of these clinical bacterial pathogens. This means that all the bacterial strains (*E.coli, S.aureus, P.aeruginosa* and *S.pneumoniae*) were resistant to any antibiotics.

**Antibacterial activities**: In this study ethanol, methanol, and n-hexane extracts of *A.afra* leaf were tested against multi-antibiotic resistant clinical pathogens. As shown in Table 2 below, the highest zone of inhibition was observed with methanolic extract (25.33±0.58 mm) against *E. coli* followed by *S.pneumoniae* (22.67±2.31mm), while the lowest zone of growth inhibition was recorded by both *P.aeruginosa* (20.00±5.00 mm) and *S. aureus* (20.00±5.00 mm) when methanolic extract was used.

**Table 2:** Antibacterial zone of growth inhibition (Mean±SD, mm) of *A.afra* leaf solvent extracts against multi-antibiotic resistant clinical isolates

	Bacterial strains			
	
Crude extracts	*E.coli*	*P.aeruginosa*	*S.aureus*	*S.pneumoniae*
**Methanolic extract**	25.33±0.58	20.00±5.00	20.00±5.00	22.67±2.31
**N-hexane extract**	10.00±0.00	11.67±7.64	5.67±1.56	11.67±2.89
**Ethanolic extract**	22.67±2.31	22.00±3.46	20.00+0.00	19.33±4.04
**DMSO**	0.00±0.00	0.00±0.00	0.00±0.00	0.00±0.00

SD: standard deviation

The highest zone of inhibition was exhibited (11.67±7.64 mm) (Table 2) when n-hexane extract was applied against *P.aeruginosa* and the lowest zone of inhibition was exhibited (5.67±1.56 mm) (Table 2) when this extract was acted against *S. aureus.*

Furthermore, zone of inhibition of ethanolic extract against selected multi-antibiotic resistant clinical pathogens was also determined and the result showed that the highest zone of inhibition was exhibited against *E.coli* (22.67±2.31mm), while the lowest zone of inhibition was exhibited (19.33±4.04 mm) (Table 2) when this extract was acted against *S.pneumoniae*.

**MIC and MBC determination**: In this study, MIC and MBC values of methanolic, ethanolic and n-hexane extracts of *A.afra* leaf were performed against those multi-antibiotic resistant clinical bacterial isolates (Table 3). The methanolic extract gave MIC value of 6.25 mg/mL for the four multi-antibiotic resistant clinical pathogens and the MIC value was 12.5 mg/mL for the pathogens *E.coli* which was 6.25 mg/mL, on the other hand, the MIC value of n-hexane extract was 25 mg/mL for all the pathogens except *E.coli*.

**Table 3:** MIC (mg/mL) and MBC (mg/mL) values of selected solvent extracts of *A.afra* leaf

MIC/MBC Values	Crude extracts	Bacterial species			

		*E.coli*	*S.aureus*	*P.aeruginosa*	*S.pneumoniae*
MIC	Methanolic extract	6.25	6.25	6.25	6.25
	n-hexane extract	12.5	25	25	25
	Ethanolic extract	6.25	12.5	12.5	12.5
MBC	Methanolic extract	6.5	12.5	12.5	12.5
	n-hexane extract	25	50	50	50
	Ethanolic extract	12.5	12.5	12.5	12.5

MIC: Minimum inhibitory concentration, MBC: Minimum bactericidal concentration

MBC value of 12.5 mg/mL was obtained when ethanol extract of *A.afra* leaf was applied against all multi-antibiotic resistant clinical bacterial isolates; however, with n-hexane extract the recorded value was 50 mg/mL against each bacterium with the exception of *E. coli* which was 25 mg/mL (Table 3). Additionally, methanolic extract of the plant leaf had MBC value of 6.25 mg/mL against *E. coli* and *P.aeruginosa*, while 12.5 mg/mL recorded against the rest isolates (Table 3).

Moreover, both from MIC and MBC values, plant extract with n-hexane had the least antibacterial effect but a good antibacterial was obtained with ethanol extract of the plant as it was determined in Table 3.

## Discussion

In this laboratory based study, the effect of crude extract of *A.afra* leaf using three solvents was tested against four multi-antibiotic resistant clinical bacterial pathogens. Initially, ten different antibiotics were tested against these bacterial strains that were taken from patient samples. All the four stains were found to be multi-antibiotic resistant. These clinical multi-antibiotic resistant bacterial isolates were used for subsequent studies.

Plants are easily accessible, less expensive, efficacious, easy to prepare, simple to use and affordable (34). Plants have been used as herbal medicine for centuries to treat infectious diseases and are considered as important sources of new antimicrobial agents (35). Some medicinal plants have been used in the making of a variety of drugs singly or in combination and even as major raw material for the production of other conventional medicines (36). Taking all these issues into consideration, *A.afra* leaf crude extract was applied against multiantibiotic resistant clinical bacterial pathogens (*E.coli, P.aeruginosa, S.aureus* and *S.pneumoniae*).

Before testing the crude extract of *A.afra*, detection of different phytochemicals this plant leaf was undertaken and it was confirmed that the crude extract of this plant leaf had several secondary metabolites with the exception of alkaloid. This means, the studied plant leaf extract contains different compounds that had contributed to its antibacterial activity against the tested multi-antibiotic resistant clinical pathogenic bacteria. Therefore, the study suggested that the crude extract of *A.afra* exhibited broad spectrum antibacterial activities against the abovementioned multi-antibiotic resistant pathogenic bacteria. Furthermore, the presence of these phytochemicals is an indicator that the plant can be potential source of precursors in the development of synthetic drugs (37). The absence of alkaloids suggests that the plant may not be toxic (37).

Then, the antibacterial effect of ethanolic, methanolic and n-hexane extracts of *A.afra* leaf were tested against each of the four multi-antibiotic resistant clinical Gram-negative (*E. coli* and *P.aeruginosa*) and Gram-positive (*S.aureus* and *S.pneumoniae*) bacterial strains. The extracts were found to have an antibacterial effect on these multi-antibiotic resistant clinical bacterial isolates. The high antibacterial activity of the methanol extract may be due to the most polar nature of the solvent, the best solvability of active ingredients in methanol, and low boiling of methanol compared to other solvents. Due to this methanol extracts exhibited higher amounts of phytochemicals.

Solvent extracts displayed higher zone of inhibition against Gram-negative bacteria compared to the Gram-positive ones. This may be because of the fact that Gram-negative bacteria are more resistant to plant secondary metabolites than Gram-positive bacteria due to the cell wall they possess linked to an outer complex membrane (murein layer), which slows down the passage of chemicals (19, 38,39). Moreover, higher zone of inhibition was exhibited on *E. coli* than that of *P.aeruginosa.* This may be because of the fact that, within the membrane of *P. aeruginosa* and not *E. coli*, three additional lipids are found as the foremost components, including phosphatidylcholine ornithine lipid and alanyl-phosphatidylglycerol that have contributed to its resistance against crude extracts of *A.afra* leaf (40).

Moreover, the antibacterial effect of all solvent extracts of *A.afra* leaf against these multi-antibiotic resistant clinical pathogens, the resistance showed on each of the test bacteria had different zone of inhibition. The difference in polarity of each solvent may probably influence the antibacterial effect of this plant.

The MIC values of the tested extracts from *A.afra* leaf against tested pathogenic bacteria were generally varied from pathogen to pathogen. The most effective minimum dilution that acted against the four bacterial strains was when methanolic and ethanolic extracts were used.

On the other hand, n-hexane extract had least mechanism of action against those multi-antibiotic resistant clinical isolates. This may be attributed to the polarity of the compounds which were extracted by each solvent and the ability of extracts to diffuse and dissolve in Mueller Hinton media.

The average zones of inhibitions recorded in the current study were better than the ones reported by Mangena *et al.* (41). This difference could be due to methodology difference, the difference in the age of the plant and difference in the solvents used for extraction. On the other hand, Keshebo *et al.* (2) recorded higher zones of inhibitions when methanolic extracts of *A.afra* leaf acted against *E.coli* 25922 and *S.aureus* 25923. The reason given for this could be the difference in concentration of the extract applied against the pathogens.

In conclusion, the *in vitro* study of antibacterial activity of *A.afra* leaf crude extract on various clinical pathogens appears rewarding as it will lead to the development of a phytomedicine to act against multiple antibiotic resistant microorganisms.
